# Right-heart reverse remodeling and heart failure symptom outcomes after catheter ablation in patients with persistent atrial fibrillation, heart failure, and functional tricuspid regurgitation

**DOI:** 10.3389/fcvm.2026.1899959

**Published:** 2026-08-07

**Authors:** Ya Shen, Peng Wang, Yihui Du, Junli Jia, Yanshan Wang

**Affiliations:** Department of Arrhythmia, Cangzhou Central Hospital, Cangzhou, Hebei, China

**Keywords:** catheter ablation, functional tricuspid regurgitation, heart failure, persistent atrial fibrillation, right-heart reverse remodeling

## Abstract

**Background:**

Persistent atrial fibrillation (AF) and heart failure (HF) may promote right-heart remodeling and functional tricuspid regurgitation (FTR). Evidence regarding right-heart changes after first AF ablation in patients with persistent AF, HF, and FTR remains limited. This study assessed 12-month right-heart reverse remodeling and its associations with HF symptoms and exploratory clinical events.

**Methods:**

This retrospective single-center cohort included 210 patients with persistent AF, HF with reduced, mildly reduced, or preserved ejection fraction, and at least mild FTR undergoing first catheter ablation. Transthoracic echocardiography (TTE) was performed at baseline and 12 months. Significant right-heart reverse remodeling was defined as a ≥10% reduction in right atrial volume index (RAVI) plus a ≥10% reduction in right ventricular (RV) basal diameter or tricuspid annular diameter, without worsening tricuspid regurgitation (TR) or RV function. Logistic regression, rhythm/loading and threshold sensitivity analyses, and 12-month landmark Cox models were used.

**Results:**

At 12 months, RAVI changed by −19.3% [interquartile range (IQR), −30.5% to −6.8%]; 93 patients (44.3%) achieved remodeling, 57.1% had TR improvement, and 61.0% had HF symptom improvement. Sinus rhythm during echocardiography increased from 16.7% to 78.6%, while heart failure guideline-directed medical therapy (HF-GDMT) and sodium-glucose cotransporter 2 (SGLT2)-inhibitor use increased. Same-rhythm and rhythm/loading-adjusted analyses showed directionally consistent but attenuated improvements in right-heart dimensions, pulmonary artery systolic pressure, and TR grade. Long-standing persistent AF, higher baseline RAVI, higher pulmonary artery systolic pressure, and arrhythmia recurrence within 12 months were associated with lower odds of remodeling, whereas higher estimated glomerular filtration rate (eGFR) and HF-GDMT were associated with higher odds. After the 12-month landmark, arrhythmia recurrence and HF rehospitalization were less frequent with remodeling.

**Conclusions:**

In this single-arm retrospective cohort, right-heart reverse remodeling observed after first AF ablation was associated with TR improvement, HF symptom improvement, and lower exploratory post-landmark event rates. The single-arm design, rhythm/loading changes, HF-GDMT intensification, and short post-landmark follow-up support associative and hypothesis-generating interpretation; causal attribution requires prospective controlled studies.

## 1 Introduction

Persistent atrial fibrillation (AF) and heart failure (HF) commonly coexist in the context of aging, obesity, hypertension, impaired renal function, and structural heart disease, and they may form mutually reinforcing pathophysiological loops through rapid or irregular ventricular response, loss of atrial mechanical function, and neurohumoral activation (1). Across the full spectrum of heart failure with preserved ejection fraction (HFpEF), heart failure with mildly reduced ejection fraction (HFmrEF), and heart failure with reduced ejection fraction (HFrEF), AF can aggravate elevated filling pressure, reduced exercise tolerance, and clinical decompensation; conversely, HF-related chamber pressure and fibrosis can maintain AF burden and promote progression of atrial cardiomyopathy (2). Functional tricuspid regurgitation (FTR), a subtype of tricuspid regurgitation (TR), is not a primary leaflet disorder but rather the result of interactions among the right atrium, tricuspid annulus, right ventricle, and pulmonary vascular loading; in the setting of AF, right atrial enlargement and tricuspid annular dilatation constitute an important anatomical substrate for atrial FTR (3). Atrial secondary TR is closely associated with long-standing AF and HFpEF, and its main mechanism is tricuspid annular dilatation and insufficient leaflet coaptation caused by right atrial enlargement; when RV dilatation, leaflet tethering, and elevated pulmonary pressure are involved, a ventricular secondary TR component may also coexist (4). FTR is associated with impaired quality of life, systemic congestion, worsening renal function, HF rehospitalization, and increased mortality risk, and its clinical significance extends beyond regurgitation grade alone (5). Assessment of TR severity requires integration of multiple parameters, including color Doppler jet characteristics, vena contracta width, proximal isovelocity surface area, regurgitant volume, hepatic venous flow, right-heart chamber size, and continuous-wave Doppler signal density; importantly, TR grade is influenced by rhythm, heart rate, and volume status (6). In patients with AF and HF, catheter-ablation-related rhythm-control strategies have been associated with lower AF burden, symptom improvement, and partial chamber reverse remodeling; evidence in the HFpEF population is increasing (7). Existing evidence on AF ablation has focused more on improvement in left ventricular ejection fraction (LVEF) and HF events, and benefits may differ across LVEF phenotypes; the relationship among right-heart structure, TR trajectory, and HF symptoms remains insufficiently characterized (8). Recent evidence in patients with AF and TR suggests that rhythm maintenance after catheter ablation may be accompanied by TR reduction and right-heart geometric changes, but the HF background, medication optimization, and composite right-heart structural endpoints have not been sufficiently integrated (9). Three-dimensional (3D) echocardiographic studies further suggest that tricuspid annular and right-heart geometry in patients with persistent AF may change in the setting of rhythm control. Therefore, evaluation of the associations among right-heart reverse remodeling, TR changes, HF symptoms, and subsequent events is warranted in the overlapping population with persistent AF, HF, and FTR (10).

## 2 Materials and methods

### 2.1 Study population

This retrospective study consecutively included patients who underwent first catheter ablation for atrial fibrillation (AF) at our hospital between December 1, 2023 and December 31, 2024 and had persistent AF, heart failure (HF), and functional tricuspid regurgitation (FTR). Inclusion criteria were as follows: (1) age ≥18 years; (2) persistent AF, defined as AF lasting >7 days or requiring pharmacological or electrical cardioversion after AF lasting ≥7 days; long-standing persistent AF was defined as continuous AF lasting ≥12 months and was recorded separately in baseline characteristics, models, and subgroup analyses (11); (3) HF diagnosed on the basis of symptoms, signs, elevated natriuretic peptides, and cardiac structural or functional abnormalities; patients were classified by left ventricular ejection fraction (LVEF) into heart failure with reduced ejection fraction (HFrEF), heart failure with mildly reduced ejection fraction (HFmrEF), and heart failure with preserved ejection fraction (HFpEF), and patients with HFpEF were additionally required to have objective evidence such as diastolic dysfunction, left atrial enlargement, left ventricular hypertrophy, or elevated pulmonary artery pressure (12); (4) at least mild and nonphysiological tricuspid regurgitation (TR) confirmed by preprocedural transthoracic echocardiography (TTE); and (5) evaluable preprocedural and 12-month clinical and echocardiographic data allowing inclusion in the 12-month clinical-echocardiographic assessment cohort.

Exclusion criteria were as follows: (1) prior AF catheter ablation or surgical maze procedure; (2) primary tricuspid valve disease, lead-related TR, previous tricuspid valve surgery, or need for concomitant valve intervention; (3) moderate-to-severe left-sided valvular stenosis or regurgitation; (4) congenital heart disease, hypertrophic cardiomyopathy, restrictive cardiomyopathy, or infiltrative cardiomyopathy; (5) acute coronary syndrome, acute myocarditis, or acute decompensated HF within 30 days before ablation; (6) severe pulmonary hypertension, defined as echocardiographically estimated pulmonary artery systolic pressure (PASP) ≥70 mmHg or severe pulmonary hypertension confirmed by right-heart catheterization; (7) chronic cor pulmonale, end-stage liver or renal dysfunction, active malignancy, active infection, or expected survival <1 year; and (8) inadequate echocardiographic image quality or missing key clinical data. After screening, 210 patients were ultimately included. The study was approved by the institutional ethics committee of our hospital. Given the retrospective nature of the study, the requirement for written informed consent was waived.

### 2.2 Clinical data collection

Baseline data within 30 days before ablation were collected from the electronic medical record system, including age, sex, body mass index (BMI), AF duration, HF duration, New York Heart Association (NYHA) functional class, blood pressure, heart rate, rhythm during echocardiography, comorbidities, CHA₂DS₂-VASc score (congestive heart failure, hypertension, age ≥75 years, diabetes mellitus, stroke or transient ischemic attack, vascular disease, age 65-74 years, and sex category), HAS-BLED score [hypertension, abnormal renal/liver function, stroke, bleeding history or predisposition, labile international normalized ratio (INR), elderly status, and drug/alcohol use], hospitalization data, and medication use.

Medication data included anticoagulants, antiarrhythmic drugs, diuretics, beta-blockers, angiotensin-converting enzyme inhibitors (ACEIs), angiotensin receptor blockers (ARBs), angiotensin receptor-neprilysin inhibitors (ARNIs), mineralocorticoid receptor antagonists (MRAs), and sodium-glucose cotransporter 2 (SGLT2) inhibitors. Heart failure guideline-directed medical therapy (HF-GDMT) was operationally defined as receipt of applicable therapeutic pillars according to HF phenotype and clinical tolerance: patients with HFrEF or HFmrEF were required to receive at least 2 disease-modifying therapeutic pillars; patients with HFpEF were required to receive an SGLT2 inhibitor and undergo volume management plus blood pressure and comorbidity optimization, or receive tolerable alternative therapy when contraindications were documented. This variable reflects receipt of phenotype-appropriate therapeutic pillars and does not indicate achievement of target doses. HF-GDMT intensification was defined as initiation, between baseline and 12 months, of at least 1 phenotype-appropriate HF therapeutic pillar or an increase of at least 50% in the dose of any preexisting therapeutic pillar. Laboratory variables were based on the most recent measurement obtained in a clinically stable state within 7 days before the procedure. Estimated glomerular filtration rate (eGFR) was calculated uniformly using the 2021 Chronic Kidney Disease Epidemiology Collaboration (CKD-EPI) creatinine equation without a race coefficient; serum creatinine was measured using a method traceable to isotope dilution mass spectrometry (IDMS) (13). All data were independently extracted and checked by 2 investigators; discrepancies were reviewed and adjudicated by a third investigator.

### 2.3 Echocardiographic assessment

All patients underwent TTE within 30 days before ablation and at 12 months after ablation. If multiple examinations were available within the same time window, the result closest to the prespecified time point and obtained during stable volume status was selected. Rhythm, heart rate, blood pressure, and volume-treatment status during the examination were recorded. Key measurements included left atrial (LA) diameter, left ventricular end-diastolic diameter (LVEDD), LVEF, right atrial volume index (RAVI), right ventricular (RV) basal diameter, tricuspid annular diameter, tricuspid annular plane systolic excursion (TAPSE), RV fractional area change, TR severity, mitral regurgitation (MR) severity, and estimated PASP. RAVI, RV basal diameter, and tricuspid annular diameter were measured in standard apical 4-chamber or RV-focused views and interpreted with adjustment for body surface area and reference ranges for right-heart geometry (14).

TR severity was assessed using an integrated approach that included the color Doppler regurgitant jet, vena contracta width, proximal isovelocity surface area (PISA), effective regurgitant orifice area, regurgitant volume, hepatic venous flow, continuous-wave Doppler signal density, and right-heart chamber size, and was graded as mild, moderate, or severe. TR improvement was defined as a decrease of ≥1 grade from baseline, and worsening was defined as an increase of ≥1 grade from baseline. FTR required exclusion of leaflet prolapse, leaflet thickening, leaflet perforation, chordal rupture, infective endocarditis, rheumatic changes, and mechanical interference caused by cardiac implantable electronic device leads. RV function was assessed using TAPSE and RV fractional area change; these measures are related to right-heart functional changes after AF ablation and were therefore recorded as part of the safety condition for the composite right-heart structural endpoint (15). Because moderate-to-severe left-sided valvular stenosis or regurgitation was an exclusion criterion, MR was analyzed descriptively only as no/trace or mild functional MR and was not a primary endpoint.

Rhythm during TTE was categorized as sinus rhythm or AF/atrial flutter (AFL), which was used to describe differences in examination status between baseline and 12 months and for rhythm/loading sensitivity analyses. Baseline and 12-month TTE images from 50 randomly selected patients were remeasured to calculate interobserver and intraobserver intraclass correlation coefficients (ICCs) and coefficients of variation (CVs) for RAVI, RV basal diameter, tricuspid annular diameter, TAPSE, and RV fractional area change. Significant right-heart reverse remodeling was defined as a ≥10% reduction from baseline in RAVI plus a ≥10% reduction in at least 1 of RV basal diameter or tricuspid annular diameter, without worsening of TR severity or RV function. The 10% threshold was prespecified to exceed the reproducibility error of 2-dimensional right-heart measurements in this study and to reduce structural endpoint misclassification caused by random fluctuation of a single parameter; this composite endpoint was interpreted as an exploratory structural endpoint.

### 2.4 Catheter ablation procedure

All patients received continuous effective anticoagulation for ≥3 weeks before the procedure. Patients with inadequate anticoagulation, poor anticoagulation adherence, or high thromboembolic risk underwent preprocedural transesophageal echocardiography (TEE) or cardiac computed tomography (CT) to exclude left atrial and left atrial appendage thrombus. Ablation was performed under local anesthesia or intravenous sedation. Transseptal puncture was performed through a femoral venous approach, and procedures were guided by a 3-dimensional electroanatomical mapping system. Open-irrigated radiofrequency ablation catheters were used. Ablation power for pulmonary vein isolation (PVI) was set at 30-45 W and adjusted according to contact force, ablation index, and disappearance of local electrograms. Catheter model, mapping system, procedure time, fluoroscopy time, and operator information were recorded. Intraprocedural heparinization was routinely used to maintain an activated clotting time (ACT) of 300-350 s.

Bilateral PVI was the fundamental ablation endpoint for all patients, and pulmonary vein entrance and exit block were confirmed; additional ablation was limited to prespecified indications, and the corresponding strategy was included in statistical adjustment (16). For patients with evidence of typical AFL or cavotricuspid isthmus (CTI)-dependent AFL, CTI linear ablation was performed and bidirectional block was confirmed. All linear ablation and posterior wall isolation procedures were recorded, and conduction block was confirmed.

Postprocedural anticoagulation was continued for at least 3 months. Thereafter, the long-term anticoagulation strategy was mainly determined by the CHA₂DS₂-VASc score and bleeding risk and was not discontinued solely because sinus rhythm was maintained. Antiarrhythmic drug use, cardioversion, and repeat ablation during the blanking period were recorded. Repeat ablation was considered an atrial arrhythmia recurrence event and analyzed separately.

### 2.5 Follow-up and study endpoints

Patients were followed at 1, 3, 6, and 12 months after ablation and every 6-12 months thereafter. The minimum follow-up duration was 12 months, and the follow-up cutoff date was December 31, 2025. Follow-up assessments included symptom evaluation, NYHA functional class, physical examination, 12-lead electrocardiogram (ECG), 24-48 h Holter monitoring, TTE, N-terminal pro-B-type natriuretic peptide (NT-proBNP), medication use, HF rehospitalization, repeat ablation, and death. Outpatient records, hospitalization records, or telephone follow-up were used to supplement data when necessary. Telephone follow-up was used only to supplement clues regarding clinical events; atrial arrhythmia recurrence required evidence from ECG or Holter monitoring. Atrial arrhythmia recurrence was defined as AF, AFL, or atrial tachycardia (AT) lasting ≥30 s after the blanking period; the recurrence-remodeling association was interpreted in the context of overlapping observation windows and potential bidirectionality (17). Rhythm-monitoring intensity was described according to scheduled 12-lead ECG completion rate, number of post-blanking Holter recordings, and cumulative Holter-monitoring duration.

The primary endpoints were percentage change in RAVI at 12 months and occurrence of significant right-heart reverse remodeling. Secondary endpoints included TR improvement, improvement in NYHA functional class, changes in NT-proBNP and LVEF, atrial arrhythmia recurrence, HF rehospitalization, and all-cause death. HF symptom improvement was defined as a decrease of at least 1 NYHA functional class from baseline at 12 months after ablation and no HF rehospitalization within 12 months after ablation. HF rehospitalization was defined as hospitalization with HF as the primary diagnosis requiring intravenous diuretics, vasoactive agents, or intensified anti-HF therapy.

To ensure correct temporal ordering when analyzing the relationship between right-heart reverse remodeling status at 12 months after ablation and subsequent clinical events, a 12-month landmark method was used, and event time was counted from completion of the 12-month assessment. In logistic models for right-heart reverse remodeling and 12-month HF symptom improvement, the atrial arrhythmia recurrence variable was restricted to recurrence recorded from after the blanking period to 12 months after ablation; recurrence after 12 months was not used to explain 12-month clinical-echocardiographic outcomes.

### 2.6 Statistical analysis

Statistical analyses were performed using SPSS 26.0 and R 4.3.2. After normality testing, normally distributed continuous variables were expressed as mean±standard deviation; between-group comparisons used the independent-samples *t* test, and paired data used the paired *t* test. Nonnormally distributed variables were expressed as median and interquartile range; between-group comparisons used the Mann–Whitney *U* test, and paired data used the Wilcoxon signed-rank test. Categorical variables were expressed as counts and percentages and compared using the *χ^2^* test or Fisher exact test. TR grade was treated as an ordinal categorical variable, and changes from before to after the procedure were analyzed using methods for paired ordinal data. Continuous variables involving multiple follow-up time points were analyzed using linear mixed models or generalized estimating equations.

Multivariable logistic regression was used to analyze factors associated with significant right-heart reverse remodeling and HF symptom improvement. Candidate variables were prespecified according to clinical relevance and included age, sex, AF duration, long-standing persistent AF, HF phenotype, baseline TR grade, RAVI, RV basal diameter, PASP, NT-proBNP, eGFR calculated using the 2021 CKD-EPI creatinine equation, HF-GDMT, ablation strategy, and atrial arrhythmia recurrence from after the blanking period to 12 months after ablation. Time-to-event outcomes were analyzed using Kaplan–Meier survival curves and Cox proportional hazards models. Odds ratios (ORs), hazard ratios (HRs), and 95% confidence intervals (CIs) were reported, and the proportional hazards assumption was tested.

Time-to-event analyses related to achievement of right-heart reverse remodeling used a 12-month landmark method; only right-heart reverse remodeling status determined at 12 months after ablation was used for event grouping, rather than using the date of ablation as the time origin. In the Cox model for HF rehospitalization, atrial arrhythmia recurrence after the 12-month landmark was treated as a time-dependent variable; because the number of HF rehospitalization events was limited, this model included only prespecified core variables.

Missing data were handled as follows: the primary structural endpoint, 12-month clinical outcomes, and candidate variables for multivariable models were complete in the final cohort of 210 patients; therefore, complete-case analysis was used for the primary analyses and multiple imputation was not performed. Candidate variables were all prespecified according to clinical relevance and were entered into the models simultaneously; no data-driven stepwise selection was used. Given the limited number of events, events per variable were reported, and Firth penalized logistic regression and ridge penalized Cox models were used as sensitivity analyses. To address potential confounding by rhythm and loading status, same-rhythm restricted analysis and rhythm/loading-adjusted models were prespecified. Because the definition of significant right-heart reverse remodeling itself included RAVI reduction and no TR worsening, RAVI change and TR worsening were described between achieved and non-achieved groups without independent between-group inferential testing. Subgroup analyses and analyses of multiple secondary endpoints were exploratory, and nominal *P* values were reported. All tests were two-sided, and *P*<0.05 was considered statistically significant.

## 3 Results

### 3.1 Study population and follow-up

**Figure 1:**
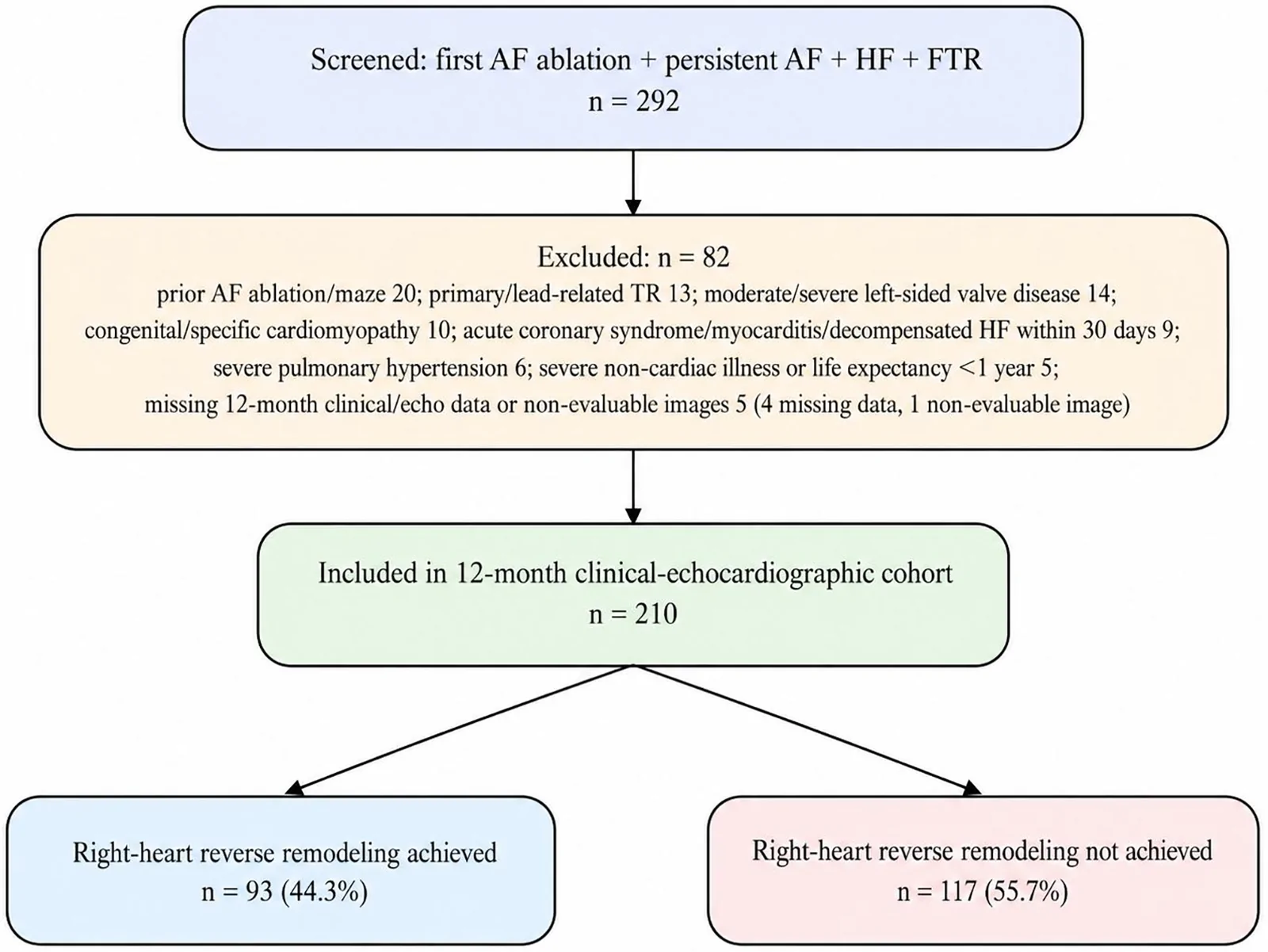
Patient screening and inclusion flow. The diagram shows 292 screened patients, 82 exclusions, and 210 patients included in the 12-month clinical-echocardiographic cohort; 5 patients were excluded because of missing 12-month clinical/echocardiographic data or non-evaluable images, including 4 with missing data and 1 with one non-evaluable image. AF, atrial fibrillation; echo, echocardiography; FTR, functional tricuspid regurgitation; HF, heart failure; RHRR, right-heart reverse remodeling; TR, tricuspid regurgitation.

A total of 292 patients were screened, and 210 patients were included in the final 12-month clinical-echocardiographic assessment cohort after 82 exclusions. The reasons for exclusion are shown in Figure 1; 5 patients were excluded because of missing 12-month clinical-echocardiographic data or non-evaluable images, including 4 with missing 12-month data and 1 with a non-evaluable image. The overall median follow-up duration was 18.3 months; the median event follow-up after the 12-month landmark was 6.1 months [interquartile range (IQR), 2.3-10.9] (Figure 1).

### 3.2 Baseline clinical characteristics

The mean age of the overall cohort was 66.9±9.6 years, and 131 patients (62.4%) were men. Long-standing persistent AF was present in 161 patients (76.7%). HFrEF, HFmrEF, and HFpEF were present in 64, 53, and 93 patients, accounting for 30.5%, 25.2%, and 44.3%, respectively. Baseline NYHA class III-IV was present in 95 patients (45.2%). At 12 months after ablation, 93 patients met the prespecified criterion for right-heart reverse remodeling, accounting for 44.3%.

Patients who achieved right-heart reverse remodeling had shorter AF duration, a lower proportion of long-standing persistent AF, lower creatinine and NT-proBNP levels, higher eGFR and albumin levels, and higher use of HF-GDMT; SGLT2 inhibitor use showed a trend toward being higher. Age, sex, BMI, HF duration, HF phenotype, NYHA functional class, blood pressure, heart rate, rhythm during echocardiography, major comorbidities, risk scores, prior HF hospitalization, length of ablation hospitalization, anticoagulants and most HF medications, hemoglobin, electrolytes, liver function, bilirubin, and troponin did not differ significantly (all *P*>0.05) (Table 1).

**Table 1:** Baseline clinical, medication, and laboratory characteristics.

Variable	Overall (n=210)	Right-heart reverse remodeling achieved (n=93)	Not achieved (n=117)	Statistic	*P* value
Age, years	66.9 ± 9.6	65.8 ± 9.2	67.8 ± 9.9	*t* = −1.51	0.132
Male sex	131 (62.4)	59 (63.4)	72 (61.5)	*χ^2^* = 0.08	0.777
Body mass index, kg/m^2^	25.3 ± 3.5	25.2 ± 3.4	25.4 ± 3.6	*t* = −0.41	0.680
AF duration, months	21 (11−46)	17 (10−35)	29 (14−55)	*Z* = −3.51	<0.001
Long−standing persistent AF	161 (76.7)	63 (67.7)	98 (83.8)	*χ^2^* = 7.43	0.006
HF duration, months	18 (8−38)	16 (7−34)	20 (9−42)	*Z* = −1.63	0.103
HF phenotype				*χ^2^* = 1.63	0.442
HFrEF	64 (30.5)	32 (34.4)	32 (27.4)		
HFmrEF	53 (25.2)	24 (25.8)	29 (24.8)		
HFpEF	93 (44.3)	37 (39.8)	56 (47.9)		
NYHA functional class				*χ^2^* = 0.94	0.624
Class II	115 (54.8)	54 (58.1)	61 (52.1)		
Class III	89 (42.4)	36 (38.7)	53 (45.3)		
Class IV	6 (2.9)	3 (3.2)	3 (2.6)		
Systolic blood pressure, mmHg	124.5 ± 15.8	123.8 ± 15.0	125.0 ± 16.4	*t* = −0.55	0.581
Diastolic blood pressure, mmHg	75.0 ± 10.2	75.3 ± 9.8	74.8 ± 10.5	*t* = 0.36	0.722
Resting heart rate, beats/min	86.0 ± 15.0	84.2 ± 14.0	87.3 ± 15.8	*t* = −1.51	0.134
AF/AFL rhythm during echocardiography	175 (83.3)	73 (78.5)	102 (87.2)	*χ^2^* = 2.81	0.093
Hypertension	138 (65.7)	58 (62.4)	80 (68.4)	*χ^2^* = 0.83	0.362
Diabetes mellitus	58 (27.6)	23 (24.7)	35 (29.9)	*χ^2^* = 0.70	0.404
Coronary artery disease	48 (22.9)	18 (19.4)	30 (25.6)	*χ^2^* = 1.16	0.281
Prior stroke/transient ischemic attack (TIA)	22 (10.5)	8 (8.6)	14 (12.0)	*χ^2^* = 0.63	0.429
eGFR <60 mL/min/1.73 m^2^	43 (20.5)	14 (15.1)	29 (24.8)	*χ^2^* = 3.01	0.083
Obstructive sleep apnea	34 (16.2)	13 (14.0)	21 (17.9)	*χ^2^* = 0.60	0.438
Chronic obstructive pulmonary disease	20 (9.5)	8 (8.6)	12 (10.3)	*χ^2^* = 0.16	0.685
CHA₂DS₂−VASc score	3 (2−4)	3 (2−4)	3 (2−4)	*Z* = −1.25	0.210
HAS−BLED score	2 (1−2)	1 (1−2)	2 (1−2)	*Z* = −1.52	0.128
HF hospitalization within the previous 12 months	61 (29.0)	23 (24.7)	38 (32.5)	*χ^2^* = 1.51	0.219
Length of ablation hospitalization, days	6 (5−8)	6 (5−7)	6 (5−8)	*Z* = −1.45	0.146
Oral anticoagulants	201 (95.7)	89 (95.7)	112 (95.7)	*χ^2^* = 0.00	0.992
Direct oral anticoagulants (DOACs)	173 (82.4)	77 (82.8)	96 (82.1)	*χ^2^* = 0.02	0.888
Warfarin	28 (13.3)	12 (12.9)	16 (13.7)	*χ^2^* = 0.03	0.870
Antiarrhythmic drugs	89 (42.4)	41 (44.1)	48 (41.0)	*χ^2^* = 0.20	0.656
Diuretics	148 (70.5)	63 (67.7)	85 (72.6)	*χ^2^* = 0.60	0.439
Beta−blockers	154 (73.3)	70 (75.3)	84 (71.8)	*χ^2^* = 0.32	0.572
ACEI/ARB/ARNI	134 (63.8)	64 (68.8)	70 (59.8)	*χ^2^* = 1.81	0.178
MRA	118 (56.2)	56 (60.2)	62 (53.0)	*χ^2^* = 1.10	0.295
SGLT2 inhibitors	97 (46.2)	49 (52.7)	48 (41.0)	*χ^2^* = 2.84	0.092
HF−GDMT	111 (52.9)	58 (62.4)	53 (45.3)	*χ^2^* = 6.06	0.014
Hemoglobin, g/L	132.0 ± 17.0	133.3 ± 16.2	131.0 ± 17.6	*t* = 0.98	0.327
Creatinine, μmol/L	87 (72−104)	83 (70−98)	91 (76−110)	*Z* = −2.27	0.023
eGFR, mL/min/1.73 m^2^	74.0 ± 21.4	78.4 ± 19.6	70.5 ± 22.2	*t* = 2.74	0.007
Albumin, g/L	40.2 ± 4.3	40.9 ± 4.1	39.6 ± 4.4	*t* = 2.21	0.028
Sodium, mmol/L	139.9 ± 3.3	140.1 ± 3.2	139.8 ± 3.4	*t* = 0.66	0.512
Potassium, mmol/L	4.12 ± 0.43	4.10 ± 0.42	4.13 ± 0.44	*t* = −0.50	0.615
Alanine aminotransferase (ALT), U/L	24 (17−36)	23 (17−35)	25 (18−37)	*Z* = −0.69	0.490
Aspartate aminotransferase (AST), U/L	26 (19−38)	25 (18−36)	27 (20−39)	*Z* = −0.89	0.373
Total bilirubin, μmol/L	15 (11−21)	14 (10−20)	16 (11−22)	*Z* = −1.51	0.132
Cardiac troponin I, μg/L	0.012 (0.006−0.024)	0.010 (0.006−0.022)	0.013 (0.007−0.026)	*Z* = −1.43	0.153
NT−proBNP, pg/mL	1440 (780−2860)	1160 (650−2190)	1720 (920−3340)	*Z* = −3.13	0.002

Continuous variables are presented as mean±standard deviation or median (interquartile range), and categorical variables are presented as n (%). Between-group comparisons for continuous variables used the independent-samples t test or Mann–Whitney U test; categorical variables were compared using the χ2 test or Fisher exact test. t denotes the t-test statistic; Z denotes the nonparametric rank-sum test statistic; χ2 denotes the chi-square test statistic; P values are two-sided. AF, atrial fibrillation; BMI, body mass index; NYHA, New York Heart Association; DOACs, direct oral anticoagulants; ACEIs, angiotensin-converting enzyme inhibitors; ARBs, angiotensin receptor blockers; ARNIs, angiotensin receptor-neprilysin inhibitors; MRAs, mineralocorticoid receptor antagonists; SGLT2, sodium-glucose cotransporter 2; eGFR, estimated glomerular filtration rate; CKD-EPI, Chronic Kidney Disease Epidemiology Collaboration; HF-GDMT, heart failure guideline-directed medical therapy; NT-proBNP, N-terminal pro-B-type natriuretic peptide.

### 3.3 Baseline echocardiographic and catheter ablation data

At baseline, mild, moderate, and severe TR were present in 68, 114, and 28 patients, accounting for 32.4%, 54.3%, and 13.3%, respectively. Patients who achieved right-heart reverse remodeling had lower baseline left atrial diameter, RAVI, RV basal diameter, tricuspid annular diameter, and PASP, and higher RV fractional area change; left ventricular end-diastolic diameter, LVEF, TAPSE, and TR grade did not differ significantly. All patients underwent PVI. Preprocedural thrombus exclusion, anesthesia approach, 3-dimensional mapping system, catheter type, ACT, procedure time, fluoroscopy time, CTI ablation, additional ablation, and operator experience did not differ significantly between groups; patients who achieved right-heart reverse remodeling had a lower proportion of cardioversion during the blanking period (Table 2).

**Table 2:** Baseline echocardiographic and catheter ablation data.

Variable	Overall (n=210)	Right-heart reverse remodeling achieved (n=93)	Not achieved (n=117)	Statistic	*P* value
Left atrial diameter, mm	48.5 ± 6.7	47.4 ± 6.2	49.4 ± 7.0	*t* = −2.19	0.029
Left ventricular end−diastolic diameter, mm	53.0 ± 7.6	52.7 ± 7.4	53.3 ± 7.8	*t* = −0.57	0.569
LVEF, %	47.6 ± 11.9	48.1 ± 11.7	47.0 ± 12.1	*t* = 0.67	0.506
RAVI, mL/m^2^	52.5 ± 13.8	49.4 ± 12.0	55.0 ± 14.7	*t* = −3.04	0.003
RV basal diameter, mm	42.4 ± 5.9	40.7 ± 5.4	43.8 ± 6.0	*t* = −3.93	<0.001
Tricuspid annular diameter, mm	39.8 ± 5.1	38.6 ± 4.8	40.8 ± 5.3	*t* = −3.15	0.002
TAPSE, mm	17.3 ± 3.2	17.6 ± 3.0	17.1 ± 3.3	*t* = 1.15	0.253
RV fractional area change, %	38.0 ± 7.2	39.3 ± 6.8	37.0 ± 7.4	*t* = 2.35	0.020
TR grade				*χ^2^* = 3.71	0.156
Mild	68 (32.4)	34 (36.6)	34 (29.1)		
Moderate	114 (54.3)	51 (54.8)	63 (53.8)		
Severe	28 (13.3)	8 (8.6)	20 (17.1)		
Estimated PASP, mmHg	42.1 ± 10.6	39.0 ± 9.1	44.5 ± 11.2	*t* = −3.93	<0.001
Preprocedural TEE or cardiac CT to exclude thrombus	79 (37.6)	31 (33.3)	48 (41.0)	*χ^2^* = 1.31	0.253
Intravenous sedation	158 (75.2)	70 (75.3)	88 (75.2)	*χ^2^* = 0.00	0.993
Three−dimensional mapping system				*χ^2^* = 0.61	0.738
CARTO	124 (59.0)	56 (60.2)	68 (58.1)		
EnSite	69 (32.9)	31 (33.3)	38 (32.5)		
Rhythmia	17 (8.1)	6 (6.5)	11 (9.4)		
Open−irrigated contact−force catheter	187 (89.0)	83 (89.2)	104 (88.9)	*χ^2^* = 0.01	0.934
Mean ACT, s	323.6 ± 22.5	323.0 ± 22.0	324.0 ± 23.0	*t* = −0.32	0.749
Procedure time, min	154.3 ± 37.6	151.0 ± 36.0	157.0 ± 39.0	*t* = −1.16	0.249
Fluoroscopy time, min	12.0 (8.0−18.0)	11.5 (8.0−17.0)	12.5 (8.0−18.5)	*Z* = −0.91	0.364
PVI completed	210 (100.0)	93 (100.0)	117 (100.0)	—	—
CTI ablation	48 (22.9)	20 (21.5)	28 (23.9)	*χ^2^* = 0.17	0.677
Any additional ablation	85 (40.5)	35 (37.6)	50 (42.7)	*χ^2^* = 0.56	0.454
Low−voltage−zone substrate ablation	55 (26.2)	22 (23.7)	33 (28.2)	*χ^2^* = 0.55	0.456
Mappable AT ablation	22 (10.5)	9 (9.7)	13 (11.1)	*χ^2^* = 0.11	0.736
Left atrial posterior wall isolation	49 (23.3)	20 (21.5)	29 (24.8)	*χ^2^* = 0.31	0.577
Non−CTI linear or critical isthmus ablation	39 (18.6)	15 (16.1)	24 (20.5)	*χ^2^* = 0.66	0.417
Operator with ≥5 years of AF ablation experience	197 (93.8)	88 (94.6)	109 (93.2)	*χ^2^* = 0.19	0.662
Antiarrhythmic drugs during the blanking period	94 (44.8)	39 (41.9)	55 (47.0)	*χ^2^* = 0.54	0.463
Electrical or pharmacological cardioversion during the blanking period	34 (16.2)	9 (9.7)	25 (21.4)	*χ^2^* = 5.22	0.022
Repeat ablation during the blanking period	0 (0)	0 (0)	0 (0)	—	—

Continuous variables are presented as mean±standard deviation or median (interquartile range), and categorical variables are presented as n (%). t denotes the independent-samples t-test statistic; Z denotes the Mann–Whitney U test statistic; χ2 denotes the chi-square test statistic; P values are two-sided. TTE, transthoracic echocardiography; LA, left atrium; LVEDD, left ventricular end-diastolic diameter; LVEF, left ventricular ejection fraction; RAVI, right atrial volume index; RV, right ventricle; TAPSE, tricuspid annular plane systolic excursion; TR, tricuspid regurgitation; PASP, pulmonary artery systolic pressure; TEE, transesophageal echocardiography; CT, computed tomography; PVI, pulmonary vein isolation; ACT, activated clotting time; CTI, cavotricuspid isthmus; AFL, atrial flutter; AT, atrial tachycardia. Additional ablation excludes PVI alone or CTI ablation alone.

### 3.4 Changes at 12 months after ablation

At 12 months after ablation, the proportion of patients in sinus rhythm increased, heart rate decreased, and favorable changes were observed in NYHA functional class, peripheral edema, jugular venous distension or hepatojugular reflux, pulmonary crackles, NT-proBNP, left atrial diameter, left ventricular end-diastolic diameter, LVEF, RAVI, RV basal diameter, tricuspid annular diameter, TAPSE, RV fractional area change, PASP, and TR grade. The proportions of patients receiving HF-GDMT and SGLT2 inhibitors increased from baseline, suggesting medication optimization during follow-up; use of antiarrhythmic drugs, diuretics, and warfarin decreased, while overall use of oral anticoagulants remained high (Table 3).

**Table 3:** Changes in clinical, medication, laboratory, and echocardiographic parameters from baseline to 12 months.

Variable	Baseline	12 months after ablation	Change	Statistic	*P* value
Systolic blood pressure, mmHg	124.5 ± 15.8	122.7 ± 14.8	−1.9 ± 13.3	*t* = −2.07	0.040
Diastolic blood pressure, mmHg	75.0 ± 10.2	73.2 ± 9.6	−1.8 ± 11.8	*t* = −2.21	0.028
Resting heart rate, beats/min	86.0 ± 15.0	71.2 ± 12.0	−14.8 ± 15.8	*t* = −13.58	<0.001
AF/AFL rhythm during echocardiography	175 (83.3)	45 (21.4)	—	McNemar *χ^2^* = 115.06	<0.001
NYHA functional class				*Z* = −9.55	<0.001
Class I	0 (0)	74 (35.2)	—		
Class II	115 (54.8)	122 (58.1)	—		
Class III	89 (42.4)	12 (5.7)	—		
Class IV	6 (2.9)	2 (1.0)	—		
Peripheral edema	66 (31.4)	24 (11.4)	—	McNemar *χ^2^* = 33.50	<0.001
Jugular venous distension or hepatojugular reflux	38 (18.1)	11 (5.2)	—	McNemar *χ^2^* = 22.88	<0.001
Pulmonary crackles	27 (12.9)	7 (3.3)	—	McNemar *χ^2^* = 16.31	<0.001
NT−proBNP, pg/mL	1440 (780−2860)	590 (320−1180)	−57.8% (−73.8% to −33.0%)	*Z* = −11.52	<0.001
Oral anticoagulants	201 (95.7)	195 (92.9)	—	McNemar *χ^2^* = 2.12	0.146
Direct oral anticoagulants (DOACs)	173 (82.4)	178 (84.8)	—	McNemar *χ^2^* = 0.77	0.380
Warfarin	28 (13.3)	17 (8.1)	—	McNemar *χ^2^* = 4.98	0.026
Antiarrhythmic drugs	89 (42.4)	60 (28.6)	—	McNemar *χ^2^* = 17.22	<0.001
Diuretics	148 (70.5)	122 (58.1)	—	McNemar *χ^2^* = 12.38	<0.001
Beta−blockers	154 (73.3)	160 (76.2)	—	McNemar *χ^2^* = 0.81	0.368
ACEI/ARB/ARNI	134 (63.8)	145 (69.0)	—	McNemar *χ^2^* = 2.89	0.089
MRA	118 (56.2)	128 (61.0)	—	McNemar *χ^2^* = 2.67	0.102
SGLT2 inhibitors	97 (46.2)	125 (59.5)	—	McNemar *χ^2^* = 13.96	<0.001
HF−GDMT	111 (52.9)	135 (64.3)	—	McNemar *χ^2^* = 10.48	0.001
Left atrial diameter, mm	48.5 ± 6.7	44.1 ± 6.4	−4.4 ± 5.5	*t* = −11.60	<0.001
Left ventricular end−diastolic diameter, mm	53.0 ± 7.6	50.7 ± 7.1	−2.3 ± 5.1	*t* = −6.54	<0.001
LVEF, %	47.6 ± 11.9	53.6 ± 10.7	6.0 ± 7.9 percentage points	*t* = 11.00	<0.001
RAVI, mL/m^2^	52.5 ± 13.8	42.4 ± 12.6	−10.1 ± 9.8	*t* = −14.94	<0.001
Percentage change in RAVI, %	—	—	−19.3 (−30.5 to −6.8)	*Z* = −11.34	<0.001
RV basal diameter, mm	42.4 ± 5.9	38.9 ± 5.6	−3.5 ± 4.6	*t* = −11.02	<0.001
Tricuspid annular diameter, mm	39.8 ± 5.1	36.8 ± 5.1	−3.0 ± 4.3	*t* = −10.11	<0.001
TAPSE, mm	17.3 ± 3.2	18.8 ± 3.1	1.5 ± 2.6	*t* = 8.36	<0.001
RV fractional area change, %	38.0 ± 7.2	42.0 ± 6.8	4.0 ± 5.9	*t* = 9.82	<0.001
Estimated PASP, mmHg	42.1 ± 10.6	35.8 ± 9.5	−6.3 ± 8.7	*t* = −10.50	<0.001
TR grade				*Z* = −10.92	<0.001
None or trace	0 (0)	36 (17.1)	—		
Mild	68 (32.4)	114 (54.3)	—		
Moderate	114 (54.3)	52 (24.8)	—		
Severe	28 (13.3)	8 (3.8)	—		
Right−heart reverse remodeling achieved	—	93 (44.3)	—	—	—

Continuous variables were analyzed using the paired t test or Wilcoxon signed-rank test; binary variables were analyzed using the McNemar test; ordinal categorical variables were analyzed using paired ordinal-data methods. t denotes the paired t-test statistic; Z denotes the Wilcoxon signed-rank test statistic; χ2 denotes the McNemar or ordinal-data test statistic; P values are two-sided. AF, atrial fibrillation; AFL, atrial flutter; NYHA, New York heart association; NT-proBNP, N-terminal pro-B-type natriuretic peptide; DOACs, direct oral anticoagulants; ACEI/ARB/ARNI, angiotensin-converting enzyme inhibitor/angiotensin receptor blocker/angiotensin receptor-neprilysin inhibitor; MRA, mineralocorticoid receptor antagonist; SGLT2, sodium-glucose cotransporter 2; HF-GDMT, heart failure guideline-directed medical therapy; LVEF, left ventricular ejection fraction; RAVI, right atrial volume index; RV, right ventricle; TAPSE, tricuspid annular plane systolic excursion; PASP, pulmonary artery systolic pressure; TR, tricuspid regurgitation.

### 3.5 Primary and secondary endpoints

**Figure 2:**
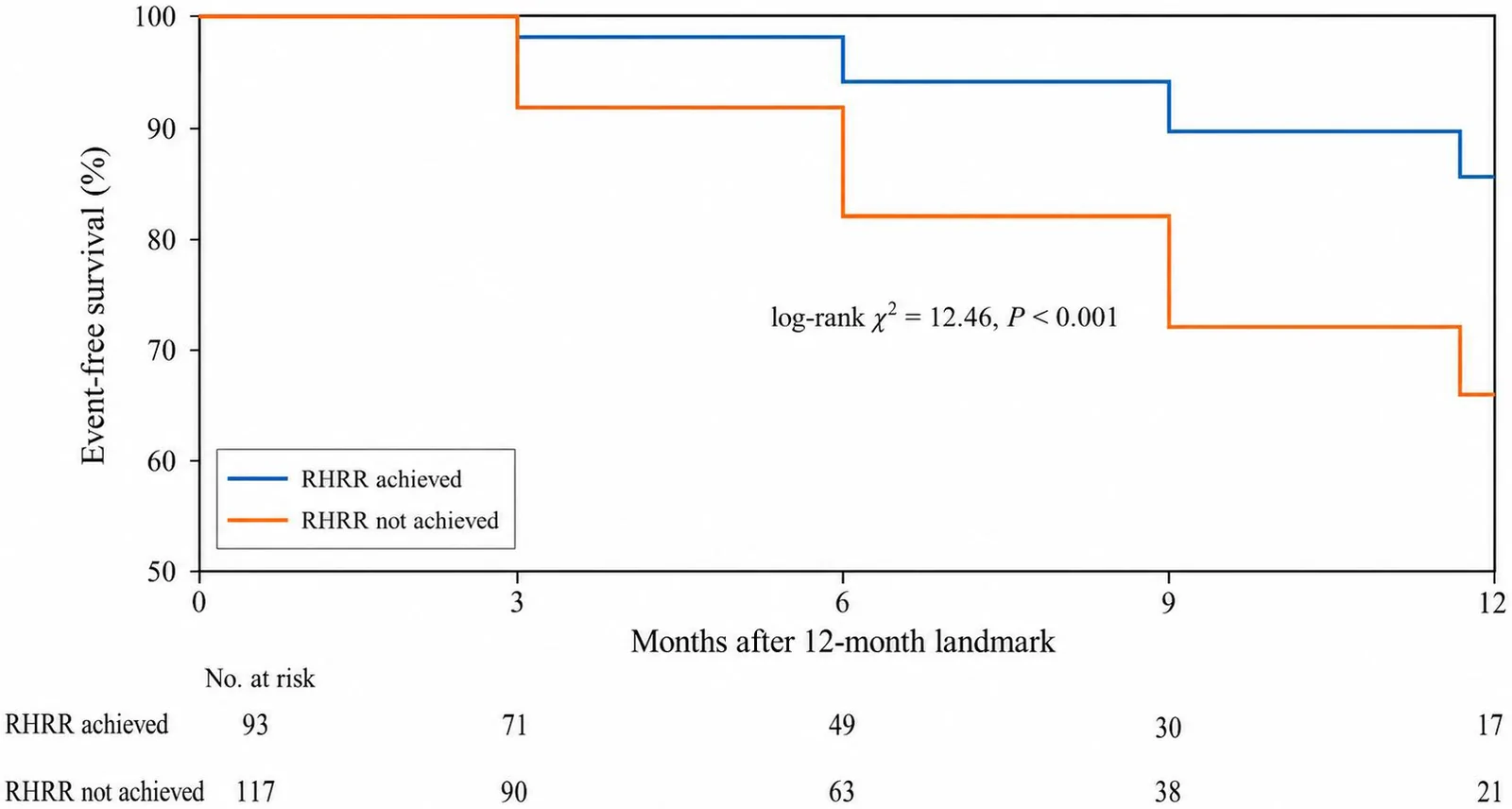
Atrial arrhythmia recurrence-free survival after the 12-month landmark. Curves start at completion of the 12-month assessment and show numbers at risk at 0, 3, 6, 9, and 12 months; between-group comparison used the log-rank test. RHRR, right-heart reverse remodeling.

**Figure 3:**
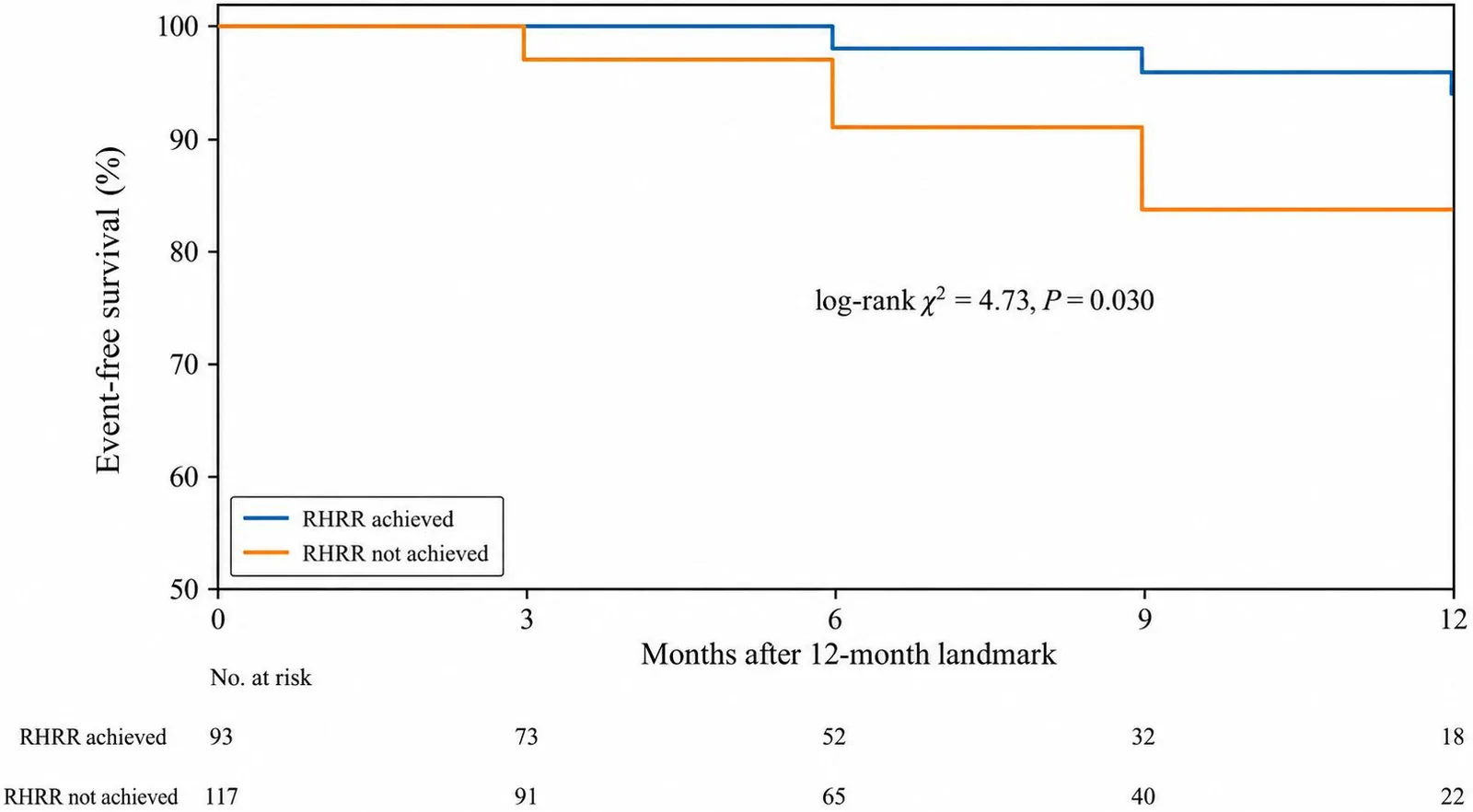
Heart failure rehospitalization-free survival after the 12-month landmark. Curves start at completion of the 12-month assessment and show numbers at risk at 0, 3, 6, 9, and 12 months; between-group comparison used the log-rank test. HF, heart failure; RHRR, right-heart reverse remodeling.

Patients who achieved right-heart reverse remodeling had higher proportions of TR improvement, improvement in NYHA functional class, and HF symptom improvement than those who did not, and had larger decreases in NT-proBNP and greater increases in LVEF. After the 12-month landmark, patients who achieved right-heart reverse remodeling had lower rates of atrial arrhythmia recurrence, AF recurrence, electrical or pharmacological cardioversion, HF rehospitalization, and the composite of HF rehospitalization or all-cause death; AFL/AT, repeat ablation, and all-cause death did not differ significantly. Because post-landmark follow-up was short and the number of events was limited, event analyses were interpreted as exploratory (Table 4, Figures 2, 3).

**Table 4:** Primary and secondary endpoints.

Variable	Overall (n=210)	Right-heart reverse remodeling achieved (n=93)	Not achieved (n=117)	Statistic	*P* value
Absolute change in RAVI, mL/m^2^	−10.1 ± 9.8	−15.8 ± 8.1	−5.5 ± 8.7	Definition−related; descriptive only	Not tested
Percentage change in RAVI, %	−19.3 (−30.5 to −6.8)	−31.0 (−39.4 to −22.2)	−9.8 (−18.0 to 2.8)	Definition−related; descriptive only	Not tested
TR improvement	120 (57.1)	78 (83.9)	42 (35.9)	*χ^2^* = 48.69	<0.001
TR worsening	6 (2.9)	0 (0)	6 (5.1)	Definition−related; descriptive only	Not tested
Improvement in NYHA functional class	136 (64.8)	73 (78.5)	63 (53.8)	*χ^2^* = 13.79	<0.001
HF symptom improvement	128 (61.0)	71 (76.3)	57 (48.7)	*χ^2^* = 16.62	<0.001
Percentage change in NT−proBNP, %	−57.8 (−73.8 to −33.0)	−63.5 (−77.6 to −44.2)	−46.5 (−66.5 to −21.5)	*Z* = −3.41	0.001
Absolute change in LVEF, percentage points	6.0 ± 7.9	7.1 ± 7.4	4.9 ± 8.2	*t* = 2.04	0.043
Atrial arrhythmia recurrence after the 12−month landmark	53 (25.2)	13 (14.0)	40 (34.2)	*χ^2^* = 11.22	0.001
AF	36 (17.1)	8 (8.6)	28 (23.9)	*χ^2^* = 8.57	0.003
AFL or AT	17 (8.1)	5 (5.4)	12 (10.3)	*χ^2^* = 1.66	0.198
Electrical or pharmacological cardioversion after the 12−month landmark	36 (17.1)	9 (9.7)	27 (23.1)	*χ^2^* = 6.55	0.010
Repeat ablation after the 12−month landmark	16 (7.6)	4 (4.3)	12 (10.3)	*χ^2^* = 2.61	0.106
HF rehospitalization after the 12−month landmark	25 (11.9)	6 (6.5)	19 (16.2)	*χ^2^* = 4.73	0.030
All−cause death after the 12−month landmark	6 (2.9)	1 (1.1)	5 (4.3)	Fisher OR = 0.24	0.231
HF rehospitalization or all−cause death after the 12−month landmark	29 (13.8)	7 (7.5)	22 (18.8)	*χ^2^* = 5.54	0.019

Categorical variables are presented as n (%), and continuous variables are presented as mean±standard deviation or median (interquartile range). χ2 denotes the chi-square test statistic; Z denotes the Mann–Whitney U test statistic; Fisher exact test was used for categorical variables with small expected cell counts; P values are two-sided. OR, odds ratio; RAVI, right atrial volume index; RV, right ventricle; TR, tricuspid regurgitation; HF, heart failure; NYHA, New York Heart Association; NT-proBNP, N-terminal pro-B-type natriuretic peptide; LVEF, left ventricular ejection fraction; AF, atrial fibrillation; AFL, atrial flutter; AT, atrial tachycardia. HF symptom improvement was defined as a decrease of at least 1 NYHA functional class from baseline at 12 months after ablation and no HF rehospitalization within 12 months after ablation. Components directly related to the definition of right-heart reverse remodeling were not subjected to independent between-group inferential testing. Clinical events after the 12-month landmark were counted from completion of the 12-month postablation assessment.

### 3.6 Multivariable logistic regression analysis

In multivariable models, long-standing persistent AF, higher RAVI, higher PASP, and atrial arrhythmia recurrence within 12 months were associated with lower odds of achieving right-heart reverse remodeling, whereas higher eGFR and HF-GDMT were associated with higher odds of achieving right-heart reverse remodeling. HF symptom improvement was associated with lower ln NT-proBNP, HF-GDMT, and no recurrence within 12 months; other candidate variables did not differ significantly (Table 5).

**Table 5:** Multivariable logistic regression analyses for achievement of right-heart reverse remodeling and heart failure symptom improvement.

Variable	Right-heart reverse remodeling achieved OR (95% CI)	Wald *χ^2^*	*P* value	HF symptom improvement OR (95% CI)	Wald *χ^2^*	*P* value
Age, per 10 years	0.90 (0.64−1.28)	0.36	0.551	0.87 (0.62−1.23)	0.64	0.426
Male sex	1.06 (0.56−2.03)	0.03	0.859	1.14 (0.60−2.16)	0.16	0.688
AF duration, per 12 months	0.92 (0.83−1.02)	2.51	0.113	0.95 (0.86−1.06)	0.92	0.336
Long−standing persistent AF	0.46 (0.23−0.92)	4.82	0.028	0.60 (0.30−1.19)	2.11	0.146
HFrEF vs HFpEF	1.20 (0.55−2.61)	0.21	0.646	1.45 (0.67−3.13)	0.89	0.345
HFmrEF vs HFpEF	1.05 (0.48−2.29)	0.01	0.903	1.18 (0.54−2.56)	0.17	0.677
Baseline moderate/severe TR vs mild TR	0.89 (0.43−1.84)	0.10	0.753	1.08 (0.53−2.18)	0.05	0.831
RAVI, per 5 mL/m^2^	0.82 (0.70−0.96)	6.07	0.014	0.94 (0.84−1.05)	1.18	0.277
RV basal diameter, per 5 mm	0.79 (0.61−1.02)	3.23	0.072	0.90 (0.72−1.13)	0.84	0.359
PASP, per 5 mmHg	0.84 (0.72−0.98)	4.91	0.027	0.89 (0.78−1.03)	2.70	0.100
ln NT−proBNP	0.78 (0.55−1.10)	1.97	0.160	0.58 (0.40−0.84)	8.28	0.004
eGFR, per 10 mL/min/1.73 m^2^	1.18 (1.02−1.37)	4.84	0.028	1.11 (0.96−1.29)	1.92	0.166
HF−GDMT	1.90 (1.03−3.51)	4.21	0.040	2.12 (1.13−3.97)	5.50	0.019
Additional ablation vs PVI ± CTI	0.94 (0.49−1.79)	0.04	0.851	1.02 (0.55−1.89)	0.00	0.950
Atrial arrhythmia recurrence from after the blanking period to 12 months after ablation	0.27 (0.13−0.58)	11.78	0.001	0.32 (0.16−0.67)	9.73	0.002

Results of multivariable logistic regression are presented as odds ratios (ORs) and 95% confidence intervals (CIs). Wald χ2 denotes the Wald test statistic; P values are two-sided. AF, atrial fibrillation; HFrEF, heart failure with reduced ejection fraction; HFmrEF, heart failure with mildly reduced ejection fraction; HFpEF, heart failure with preserved ejection fraction; TR, tricuspid regurgitation; NT-proBNP, N-terminal pro-B-type natriuretic peptide; RAVI, right atrial volume index; RV, right ventricle; PASP, pulmonary artery systolic pressure; eGFR, estimated glomerular filtration rate; HF-GDMT, heart failure guideline-directed medical therapy; PVI, pulmonary vein isolation; CTI, cavotricuspid isthmus; ln, natural logarithm. NT-proBNP was entered into the model after natural-log transformation. Variables entered into the model were prespecified according to clinical relevance. The atrial arrhythmia recurrence variable was restricted to recurrences from after the blanking period to 12 months after ablation.

### 3.7 Post-landmark Cox models

The post-landmark model for atrial arrhythmia recurrence showed that long-standing persistent AF and larger left atrial diameter were associated with higher recurrence risk. The HF rehospitalization model showed that post-landmark recurrence and higher ln NT-proBNP were associated with higher rehospitalization risk, whereas higher eGFR and HF-GDMT were associated with lower rehospitalization risk; because the number of events was limited, this model was interpreted as exploratory (Table 6).

**Table 6:** Cox proportional hazards models for time-to-event outcomes after the 12-month landmark.

Outcome and variable	HR (95% CI)	Wald *χ^2^*	*P* value
Atrial arrhythmia recurrence after the 12−month landmark			
Age, per 10 years	1.10 (0.79−1.52)	0.33	0.568
Long−standing persistent AF	1.93 (1.03−3.62)	4.21	0.040
Baseline left atrial diameter, per 5 mm	1.27 (1.03−1.57)	4.94	0.026
HFrEF vs HFpEF	1.05 (0.55−2.00)	0.02	0.882
HFmrEF vs HFpEF	0.93 (0.45−1.91)	0.04	0.844
Additional ablation vs PVI±CTI	1.18 (0.66−2.12)	0.31	0.578
Baseline moderate/severe TR vs mild TR	1.13 (0.58−2.19)	0.13	0.718
HF−GDMT	0.80 (0.45−1.42)	0.58	0.447
HF rehospitalization after the 12−month landmark			
Atrial arrhythmia recurrence, time−dependent variable	2.86 (1.36−6.02)	7.67	0.006
ln NT−proBNP	1.66 (1.10−2.50)	5.86	0.016
eGFR, per 10 mL/min/1.73 m^2^	0.80 (0.64−0.99)	4.02	0.045
PASP, per 5 mmHg	1.13 (0.97−1.32)	2.42	0.120
HF−GDMT	0.48 (0.23−0.99)	3.89	0.049

Time-to-event analyses used Cox proportional hazards models, and results are presented as hazard ratios (HRs) and 95% confidence intervals (CIs). Wald χ2 denotes the Wald test statistic; P values are two-sided. AF, atrial fibrillation; LA, left atrium; HFrEF, heart failure with reduced ejection fraction; HFmrEF, heart failure with mildly reduced ejection fraction; HFpEF, heart failure with preserved ejection fraction; TR, tricuspid regurgitation; PVI, pulmonary vein isolation; CTI, cavotricuspid isthmus; NT-proBNP, N-terminal pro-B-type natriuretic peptide; eGFR, estimated glomerular filtration rate; PASP, pulmonary artery systolic pressure; HF-GDMT, heart failure guideline-directed medical therapy; ln, natural logarithm. Analyses used completion of the 12-month assessment as the landmark starting point. Because the number of HF rehospitalization events was limited, the HF rehospitalization model included only prespecified core variables; atrial arrhythmia recurrence was treated as a time-dependent variable. Because all-cause death events were few, only descriptive statistics were performed and no multivariable Cox model was applied.

### 3.8 Supplementary analyses

Rhythm/loading sensitivity analyses, threshold sensitivity analyses, measurement reproducibility, penalized models, monitoring intensity, and descriptive analysis of mild functional MR all supported the direction of the main analysis. Subgroup analyses according to AF duration, HF phenotype, and baseline TR grade were exploratory; patients with non-long-standing persistent AF had higher proportions of right-heart reverse remodeling, TR improvement, and HF symptom improvement; main right-heart and event endpoints did not differ significantly across HF phenotypes; and TR improvement was more common in patients with moderate/severe TR (Supplementary Tables S1-S8).

## 4 Discussion

In this overlapping population with persistent AF, HF, and FTR, favorable changes in right-heart structure and symptom status were observed at 12 months. Right-heart reverse remodeling occurred in parallel with TR improvement, NT-proBNP reduction, LVEF increase, and HF symptom improvement, suggesting that right-heart structural status may be an important dimension of comprehensive postablation assessment (18). Compared with recent studies related to persistent AF and FTR, the novelty of the present study is that it included the full spectrum of HF phenotypes by LVEF and integrated HF-GDMT, a composite right-heart structural endpoint, TR changes, HF symptoms, recurrence within 12 months, and events after a 12-month landmark within the same analytic framework (19). Baseline TR and right-heart structural abnormalities may represent an integrated phenotype of AF-maintaining substrate and right-heart pressure-volume loading. TR severity has been associated with subsequent AF recurrence risk, suggesting that right-heart and tricuspid structural status may participate in the pathophysiological process underlying difficulty in rhythm maintenance (20). Atrial FTR and ventricular FTR may coexist in this cohort. Long-standing AF-related right atrial enlargement and tricuspid annular dilatation constitute the substrate of atrial FTR; HF, elevated pulmonary pressure, and RV dilatation may contribute to a ventricular FTR component through leaflet tethering, papillary muscle displacement, and altered RV geometry (21). Right-heart reverse remodeling was more common in patients with less right-heart enlargement and lower pulmonary vascular loading, suggesting that reversibility may depend on the balance among atrial-annular coupling, RV geometric changes, and pulmonary vascular pressure loading. When the atrial phenotype is accompanied by relatively preserved RV geometry, reversibility after rhythm maintenance and volume optimization may be greater (22). Ventricular secondary TR or mixed TR may limit right-heart structural recovery. In this study, higher PASP and larger RV basal diameter were associated with remodeling failure, consistent with the mechanistic influence of ventricular-pulmonary vascular loading on the reversibility of TR (23). Longer AF duration was associated with lower probability of right-heart reverse remodeling, supporting the clinical value of early identification of persistent AF, right atrial enlargement, and FTR. Risk factors for AF-related TR are intertwined with atrioventricular structural remodeling, HF status, and pulmonary vascular pressure loading, and right-heart outcome differences cannot be fully explained by a single rhythm intervention (24). Higher left atrial pressure and left-heart loading can influence the right heart and TR through pulmonary vascular pressure transmission. Left atrial enlargement, functional MR, and elevated pulmonary vascular pressure may form a left-heart-pulmonary vascular-right-heart loading pathway, thereby affecting FTR severity and right-heart reversibility (25). Renal function was associated with right-heart reverse remodeling and HF rehospitalization risk, suggesting that volume-management capacity, venous congestion, cardiorenal interaction, and medication tolerance may jointly affect right-heart recovery. The relationship between renal function trajectory after AF ablation and clinical outcomes should be interpreted in the context of baseline chronic kidney disease status (26). The proportions of patients receiving HF-GDMT and SGLT2 inhibitors increased during follow-up, and medication optimization constitutes an important co-explanation for imaging and symptom improvement. GDMT may reduce filling pressures, improve volume status, alleviate pulmonary vascular loading, and promote anti-remodeling of the left and right heart, thereby providing a synergistic background with rhythm maintenance (27). Left atrial remodeling and changes in mild functional MR provide an additional pathway for interpreting right-heart improvement. AF-related left atrial enlargement may cause annular enlargement and functional MR; when left atrial size and MR burden decrease, pulmonary vascular pressure and right-heart loading may improve in parallel (28). Outcomes after AF ablation in patients with HFpEF are influenced by left atrial disease, diastolic function, volume load, and comorbidities. The present study covered the full spectrum of HF phenotypes, and right-heart remodeling did not differ significantly across HF phenotypes, suggesting that recovery of right-heart structure may be more strongly influenced by AF duration, right-heart loading, renal function, and GDMT status (29). In patients with AF and a cardiomyopathy phenotype, LVEF improvement after rhythm control may reflect the combined effects of reduced arrhythmia burden, ventricular rate control, and medication optimization. In this study, the magnitude of LVEF increase was greater in HFrEF, but right-heart reverse remodeling did not differ significantly across HF phenotypes (30). TR reduction and right-heart geometric improvement after rhythm restoration are clinically meaningful at the individual-patient level. However, TR grade is sensitive to rhythm, heart rate, and volume status at the time of examination; the rhythm/loading sensitivity analyses in this study showed consistent directions but smaller effect sizes, supporting cautious interpretation (31). FTR with HF may constitute a distinct clinical phenotype. Right atrial enlargement, tricuspid annular dilatation, RV geometric changes, elevated PASP, and venous congestion can amplify one another and thereby influence HF symptoms and rehospitalization risk (32). Functional MR and FTR may share a common substrate of atrial disease and annular dilatation. Improvement in mild MR may indirectly affect the right heart by reducing left atrial and pulmonary vascular loading; after exclusion of moderate-to-severe left-sided valvular disease, this mechanism may still provide an auxiliary explanation for right-heart changes (33). The effects of rhythm control on MR and TR should be interpreted within a whole-heart remodeling framework. Left and right atrial dimensions, bilateral annular geometry, pulmonary vascular pressure, and GDMT status jointly determine the reversibility of valvular regurgitation (34). Overall, this study supports expanding postablation management in patients with persistent AF, HF, and FTR from rhythm maintenance alone to comprehensive assessment of rhythm status, right-heart structure, TR, left atrium and mild functional MR, volume status, renal function, and HF-GDMT (35).

This study has several limitations. The data were derived from a single-center retrospective cohort, and the primary structural endpoint required completion of 12-month TTE and clinical assessment, which may have introduced completer-cohort bias and may have overestimated the proportions of right-heart reverse remodeling, TR improvement, and HF symptom improvement, thereby limiting generalizability to patients with early death, loss to follow-up, or clinical instability. Rhythm, heart rate, and volume status at the time of TTE differed systematically between examinations, and although sensitivity analyses showed consistent directions, residual uncertainty remains regarding the relative contributions of acute loading changes and chronic structural remodeling. HF-GDMT and SGLT2 inhibitor use increased during follow-up, and medication optimization is an important co-explanation for imaging and symptom improvement. Post-landmark follow-up was short and the number of events was limited; event analyses were used only to generate hypotheses for future studies. Intermittent Holter monitoring may have underestimated asymptomatic recurrence; the relationship between recurrence and 12-month remodeling status may also be bidirectional. Some patients lacked 3D echocardiography, cardiac magnetic resonance imaging, or invasive hemodynamic validation, and the composite right-heart reverse remodeling endpoint requires external validation.

## 5 Conclusions

In patients with persistent AF, HF, and FTR, the 12-month assessment after first catheter ablation showed favorable changes in right-heart structure, RV function, TR, NT-proBNP, and NYHA functional class. Shorter AF duration, less right-heart enlargement and pulmonary vascular loading, better renal function, HF-GDMT, and rhythm maintenance were associated with right-heart reverse remodeling and symptom improvement. Patients who achieved right-heart reverse remodeling subsequently had lower rates of atrial arrhythmia recurrence and HF rehospitalization; given the single-center retrospective single-arm design, short post-landmark follow-up, and limited number of events, these event findings represent exploratory associations. Postablation management should comprehensively assess rhythm status, right-heart structure, TR, left atrium and mild functional MR, volume status, renal function, and HF-GDMT.

## Data Availability

The original contributions presented in the study are included in the article/Supplementary Material, further inquiries can be directed to the corresponding author.
