# Supplementary material for: Right-heart reverse remodeling and heart failure symptom outcomes after catheter ablation in patients with persistent atrial fibrillation, heart failure, and functional tricuspid regurgitation
Source: Front Cardiovasc Med. 2026 Aug 7;13:1899959. doi: 10.3389/fcvm.2026.1899959 (PMC13493284; doi:10.3389/fcvm.2026.1899959)
Supplement: Supplementary file 1 [file Datasheet1.docx]

# Supplementary Material

**Supplementary Table 1. Cross-tabulation of rhythm during baseline and 12-month transthoracic echocardiography**

| **Baseline TTE rhythm** | **Sinus rhythm at 12 months** | **AF/AFL at 12 months** | **Total** |
| --- | --- | --- | --- |
| Sinus rhythm | 27 (12.9) | 8 (3.8) | 35 (16.7) |
| AF/AFL | 138 (65.7) | 37 (17.6) | 175 (83.3) |
| Total | 165 (78.6) | 45 (21.4) | 210 (100.0) |

Note: TTE, transthoracic echocardiography; AF, atrial fibrillation; AFL, atrial flutter. Categorical variables are presented as n (%). This table is a descriptive rhythm cross-tabulation and was not subjected to between-group inferential testing.

**Supplementary Table 2. Rhythm/loading sensitivity analyses**

| **Analysis** | **Parameter** | **Value** | **Statistical method** | ***P* value** | **Interpretation** |
| --- | --- | --- | --- | --- | --- |
| Same-rhythm subgroup (n=64) | RAVI, mL/m² | 50.9±13.2 to 43.5±12.7; change −7.4±8.9 | Paired *t* test | <0.001 | Effect size was conservatively attenuated relative to the main analysis |
| Same-rhythm subgroup (n=64) | RV basal diameter, mm | 41.8±5.7 to 39.3±5.6; change −2.5±4.0 | Paired *t* test | <0.001 | Directionally consistent with the main analysis |
| Same-rhythm subgroup (n=64) | Tricuspid annular diameter, mm | 39.4±4.9 to 37.3±5.0; change −2.1±3.8 | Paired *t* test | <0.001 | Directionally consistent with the main analysis |
| Same-rhythm subgroup (n=64) | PASP, mmHg | 41.2±10.4 to 36.6±9.6; change −4.6±8.4 | Paired *t* test | <0.001 | Directionally consistent with the main analysis |
| Same-rhythm subgroup (n=64) | TR improvement | 30/64 (46.9%) | Paired ordinal-data test | <0.001 | The improvement proportion was lower than in the overall cohort but directionally consistent |
| Adjusted model (n=210) | Time effect for RAVI | β=−8.1 mL/m² (95% CI, −9.7 to −6.5) | Linear mixed model | <0.001 | Adjusted for rhythm, heart rate, blood pressure, diuretic use, and HF-GDMT |
| Adjusted model (n=210) | Time effect for TR grade improvement | OR=2.68 (95% CI, 1.80-3.98) | GEE ordinal logistic model | <0.001 | Adjusted for rhythm/loading variables |

Note: The paired *t* test was used for before-after comparisons of continuous variables in the same-rhythm subgroup; a paired ordinal-data test was used for TR grade changes; linear mixed models and generalized estimating equations (GEEs) were used for sensitivity analyses adjusted for rhythm, heart rate, blood pressure, diuretic use, and HF-GDMT status at echocardiography. OR, odds ratio; CI, confidence interval; RAVI, right atrial volume index; RV, right ventricle; PASP, pulmonary artery systolic pressure; TR, tricuspid regurgitation; HF-GDMT, heart failure guideline-directed medical therapy.

**Supplementary Table 3. Reproducibility of right-heart echocardiographic measurements**

| **Parameter** | **Interobserver ICC (95% CI)** | **Intraobserver ICC (95% CI)** | **Interobserver CV** | **Intraobserver CV** |
| --- | --- | --- | --- | --- |
| RAVI | 0.93 (0.90-0.96) | 0.96 (0.94-0.98) | 5.8% | 4.3% |
| RV basal diameter | 0.91 (0.87-0.94) | 0.94 (0.91-0.96) | 5.2% | 3.9% |
| Tricuspid annular diameter | 0.89 (0.84-0.92) | 0.93 (0.90-0.95) | 6.4% | 4.7% |
| TAPSE | 0.92 (0.88-0.95) | 0.95 (0.92-0.97) | 5.0% | 3.6% |
| RV fractional area change | 0.88 (0.83-0.92) | 0.92 (0.88-0.95) | 6.7% | 5.4% |

Note: ICC, intraclass correlation coefficient; CV, coefficient of variation; CI, confidence interval; RAVI, right atrial volume index; RV, right ventricle; TAPSE, tricuspid annular plane systolic excursion; TTE, transthoracic echocardiography. ICCs were estimated using a two-way random-effects model.

**Supplementary Table 4. Threshold sensitivity analysis for right-heart reverse remodeling**

| **Threshold definition** | **Remodeling achieved** | **HF symptom improvement** | **Post-landmark atrial arrhythmia recurrence** | **Post-landmark HF rehospitalization** |
| --- | --- | --- | --- | --- |
| 5% threshold | 118/210 (56.2%) | 86/118 (72.9%) vs 42/92 (45.7%), *P* < 0.001 | 20/118 (16.9%) vs 33/92 (35.9%), *P* = 0.002 | 10/118 (8.5%) vs 15/92 (16.3%), *P* = 0.082 |
| 10% threshold (main analysis) | 93/210 (44.3%) | 71/93 (76.3%) vs 57/117 (48.7%), *P* < 0.001 | 13/93 (14.0%) vs 40/117 (34.2%), *P* = 0.001 | 6/93 (6.5%) vs 19/117 (16.2%), *P* = 0.030 |
| 15% threshold | 66/210 (31.4%) | 51/66 (77.3%) vs 77/144 (53.5%), *P* = 0.001 | 8/66 (12.1%) vs 45/144 (31.3%), *P* = 0.003 | 4/66 (6.1%) vs 21/144 (14.6%), *P* = 0.077 |

Note: Threshold sensitivity analyses used 5%, 10%, and 15% thresholds for right-heart reverse remodeling. Between-group categorical variables were compared using the *χ²* test or Fisher exact test; *P* values are two-sided. HF, heart failure. The 10% threshold was the main-analysis definition.

**Supplementary Table 5. Events per variable and penalized/parsimonious models**

| **Model** | **Number of events/minority outcome count** | **Number of parameters or covariates** | **EPV** | **Key results of sensitivity model** |
| --- | --- | --- | --- | --- |
| Right-heart reverse remodeling logistic model | 93 | 15 | 6.2 | Firth: long-standing persistent AF OR=0.50, *P* = 0.035; RAVI OR=0.84, *P* = 0.028; HF-GDMT OR=1.78, *P* = 0.039; recurrence within 12 months OR=0.31, *P* = 0.001 |
| HF symptom improvement logistic model | 82 | 15 | 5.5 | Firth: ln NT-proBNP OR=0.61, *P* = 0.004; HF-GDMT OR=2.01, *P* = 0.016; recurrence within 12 months OR=0.34, *P* = 0.002 |
| Post-landmark atrial arrhythmia recurrence Cox model | 53 | 8 | 6.6 | Ridge Cox: long-standing persistent AF HR=1.78, *P* = 0.050; left atrial diameter per 5 mm HR=1.22, *P* = 0.048 |
| Post-landmark HF rehospitalization Cox model | 25 | 5 | 5.0 | Ridge Cox: time-dependent recurrence HR=2.55, *P* = 0.010; ln NT-proBNP HR=1.52, *P* = 0.027; eGFR HR=0.83, *P* = 0.055; HF-GDMT HR=0.55, *P* = 0.094 |

Note: EPV, events per variable; OR, odds ratio; HR, hazard ratio; CI, confidence interval; Cox, Cox proportional hazards model; AF, atrial fibrillation; HF, heart failure; NT-proBNP, N-terminal pro-B-type natriuretic peptide; RAVI, right atrial volume index; PASP, pulmonary artery systolic pressure; eGFR, estimated glomerular filtration rate; HF-GDMT, heart failure guideline-directed medical therapy. Firth penalized logistic regression and ridge penalized Cox models were used to evaluate the robustness of model estimates when event counts were limited.

**Supplementary Table 6. Rhythm-monitoring intensity and adherence**

| **Monitoring metric** | **Overall** | **Right-heart reverse remodeling achieved** | **Not achieved** |
| --- | --- | --- | --- |
| Scheduled 12-lead ECG completion rate | 806/840 (96.0%) | 358/372 (96.2%) | 448/468 (95.7%) |
| At least 1 post-blanking Holter recording | 205/210 (97.6%) | 91/93 (97.8%) | 114/117 (97.4%) |
| At least 2 post-blanking Holter recordings | 187/210 (89.0%) | 84/93 (90.3%) | 103/117 (88.0%) |
| At least 3 post-blanking Holter recordings | 132/210 (62.9%) | 61/93 (65.6%) | 71/117 (60.7%) |
| Cumulative Holter duration within 12 months after ablation | 96 h (72-120) | 96 h (72-120) | 96 h (72-120) |
| At least 1 Holter recording after the landmark | 156/210 (74.3%) | 72/93 (77.4%) | 84/117 (71.8%) |
| Cumulative Holter duration after the landmark | 48 h (24-72) | 48 h (24-72) | 48 h (24-72) |

Note: ECG, electrocardiogram; Holter, Holter monitoring; IQR, interquartile range. Categorical variables are presented as n/N (%), and nonnormally distributed continuous variables are presented as median (interquartile range). This table describes rhythm-monitoring intensity and adherence and was not subjected to between-group inferential testing. Because post-landmark follow-up was short and the number of events was limited, the corresponding time-to-event analyses were interpreted as exploratory.

**Supplementary Table 7. Descriptive analysis of mild functional mitral regurgitation**

| **Parameter** | **Baseline** | **12 months** | **Change/event** | **Statistical method** | ***P* value** |
| --- | --- | --- | --- | --- | --- |
| No/trace MR | 82 (39.0) | 112 (53.3) | +30 patients | McNemar test | <0.001 |
| Mild functional MR | 128 (61.0) | 98 (46.7) | −30 patients | McNemar test | <0.001 |
| MR reduction ≥1 grade | — | — | 35 (16.7) | Descriptive statistics | — |
| MR increase by 1 grade | — | — | 5 (2.4) | Descriptive statistics | — |
| Left atrial diameter | 48.5±6.7 mm | 44.1±6.4 mm | −4.4±5.5 mm | Paired *t* test | <0.001 |

Note: MR, mitral regurgitation; the McNemar test was used for paired binary variables; the paired *t* test was used for before-after comparison of left atrial diameter; *P* values are two-sided. The descriptive analysis of mild functional MR was limited to the cohort after exclusion of moderate-to-severe left-sided valvular disease.

**Supplementary Table 8. Prespecified subgroup analyses**

| **Subgroup** | **n** | **Right-heart reverse remodeling achieved** | **TR improvement** | **HF symptom improvement** | **Atrial arrhythmia recurrence after the 12-month landmark** | **HF rehospitalization after the 12-month landmark** | **Absolute change in LVEF, percentage points** |
| --- | --- | --- | --- | --- | --- | --- | --- |
| Stratified by AF duration |  |  |  |  |  |  |  |
| Persistent AF <12 months | 49 | 30 (61.2) | 34 (69.4) | 36 (73.5) | 6 (12.2) | 4 (8.2) | 6.7±7.7 |
| Long-standing persistent AF | 161 | 63 (39.1) | 86 (53.4) | 92 (57.1) | 47 (29.2) | 21 (13.0) | 5.8±8.0 |
| Statistic |  | *χ²*=7.43 | *χ²*=3.91 | *χ²*=4.21 | *χ²*=5.72 | *χ²*=0.85 | *t*=0.71 |
| *P* value |  | 0.006 | 0.048 | 0.040 | 0.017 | 0.356 | 0.480 |
| Stratified by HF phenotype |  |  |  |  |  |  |  |
| HFrEF | 64 | 32 (50.0) | 39 (60.9) | 44 (68.8) | 15 (23.4) | 9 (14.1) | 11.6±8.6 |
| HFmrEF | 53 | 24 (45.3) | 30 (56.6) | 33 (62.3) | 13 (24.5) | 6 (11.3) | 6.4±6.5 |
| HFpEF | 93 | 37 (39.8) | 51 (54.8) | 51 (54.8) | 25 (26.9) | 10 (10.8) | 1.8±5.0 |
| Statistic |  | *χ²*=1.63 | *χ²*=0.58 | *χ²*=3.13 | *χ²*=0.26 | *χ²*=0.42 | *F*=41.32 |
| *P* value |  | 0.442 | 0.747 | 0.209 | 0.879 | 0.811 | <0.001 |
| Stratified by baseline TR grade |  |  |  |  |  |  |  |
| Mild | 68 | 34 (50.0) | 21 (30.9) | 43 (63.2) | 15 (22.1) | 6 (8.8) | 5.5±7.6 |
| Moderate | 114 | 51 (44.7) | 76 (66.7) | 71 (62.3) | 30 (26.3) | 14 (12.3) | 6.1±8.1 |
| Severe | 28 | 8 (28.6) | 23 (82.1) | 14 (50.0) | 8 (28.6) | 5 (17.9) | 6.5±7.9 |
| Statistic |  | *χ²*=3.71 | *χ²*=30.52 | *χ²*=1.64 | *χ²*=0.60 | *χ²*=1.58 | *F*=0.20 |
| *P* value |  | 0.156 | <0.001 | 0.439 | 0.741 | 0.455 | 0.821 |

Note: Categorical variables are presented as n (%), and continuous variables are presented as mean±standard deviation. The *χ²* test was used for two-group or multigroup categorical comparisons; the independent-samples *t* test or one-way analysis of variance was used for continuous variables; *P* values are two-sided. AF, atrial fibrillation; TR, tricuspid regurgitation; HF, heart failure; LVEF, left ventricular ejection fraction; HFrEF, heart failure with reduced ejection fraction; HFmrEF, heart failure with mildly reduced ejection fraction; HFpEF, heart failure with preserved ejection fraction. Atrial arrhythmia recurrence and HF rehospitalization refer to events after the 12-month landmark. LVEF change represents the absolute change in LVEF in percentage points. Subgroup analyses were exploratory and nominal *P* values are reported. *F* denotes the one-way analysis-of-variance statistic.
